# Social Media for the Dissemination of Cochrane Child Health Evidence: Evaluation Study

**DOI:** 10.2196/jmir.7819

**Published:** 2017-09-01

**Authors:** Michele P Dyson, Amanda S Newton, Kassi Shave, Robin M Featherstone, Denise Thomson, Aireen Wingert, Ricardo M Fernandes, Lisa Hartling

**Affiliations:** ^1^ Alberta Research Centre for Health Evidence University of Alberta Edmonton, AB Canada; ^2^ Department of Pediatrics University of Alberta Edmonton, AB Canada; ^3^ Cochrane Child Health University of Alberta Edmonton, AB Canada; ^4^ Faculty of Medicine Universidade de Lisboa Lisbon Portugal; ^5^ Cochrane Portugal Universidade de Lisboa Lisbon Portugal

**Keywords:** social media, translational medical research, health knowledge, attitudes, practice, pediatrics

## Abstract

**Background:**

Health care providers value ready access to reliable synthesized information to support point-of-care decision making. Web-based communities, facilitated by the adoption of social media tools such as Facebook, Twitter, and YouTube, are increasingly being used for knowledge dissemination, bridging the gap between knowledge generation and synthesis and knowledge implementation.

**Objective:**

Our objective was to implement and evaluate a structured social media strategy, using multiple platforms, to disseminate Cochrane Child Health evidence to health care providers caring for children.

**Methods:**

Our social media strategy had three components: daily “tweets” using the Cochrane Child Health Twitter account, weekly WordPress blog posts, and a monthly journal club on Twitter (“tweet chat”). Each tweet, blog, and journal club shared Cochrane evidence on a child health topic. We evaluated the strategy through (1) Twitter and blog site analytics, (2) traceable link (Bitly) statistics, (3) Altmetric.com scores for promoted evidence, and (4) participant feedback. We also tracked the resources required to write the blog, tweet content, and manage the strategy.

**Results:**

The 22-week social media strategy ran between November 2014 and April 2015. We created 25 blog posts, sent 585 tweets, and hosted 3 tweet chats. Monthly blog visits and views and Twitter account followers increased over time. During the study period, the blog received 2555 visitors and 3967 page views from a geographically diverse audience of health care providers, academics, and health care organizations. In total, 183 traceable Bitly links received 3463 clicks, and the Twitter account gained 469 new followers. The most visited and viewed blog posts included gastrointestinal topics (lactose avoidance), research on respiratory conditions (honey for cough and treatments for asthma), and maternal newborn care (skin-to-skin contact). On Twitter, popular topics were related to public health (vaccination) and pain management. We collected Altmetric.com scores for 61 studies promoted during the study period and recorded an average increase of 11 points. Research staff (n=3) contributed approximately 433 hours to promotion activities and planning (6.5 hours each per week) to implement the social media strategy, and study investigators reviewed all content (blog posts and tweets).

**Conclusions:**

This study provides empirical evidence on the use of a coordinated social media strategy for the dissemination of evidence to professionals providing health services to children and youth. The results and lessons learned from our study provide guidance for future knowledge dissemination activities using social media tools.

## Introduction

Advances in technology have markedly changed the way individuals can access and use information. The use of social media and Web 2.0 technologies is rapidly changing the health landscape, redefining the way health care providers connect professionally with colleagues and patients [1,2]. Social media tools such as Facebook, Twitter, and YouTube are increasingly being used by health care providers to access virtual communities where research evidence can be shared and exchanged [3-6]. Please see Multimedia Appendix 1 for a glossary of social media terms. The Web-based interface of social media transcends many traditional geographic barriers, creating the potential for connecting health care providers who might not otherwise interact [7]. Health care providers value ready access to highly synthesized and reliable information to support point-of-care decision making. The wide reach and accessibility of social media create opportunities for expanded dissemination of evidence among professional health care networks and ultimately increase opportunities for the uptake and implementation of evidence in practice [8].

Social media may have great potential for use as a rapid, accessible, and cost-effective strategy to disseminate knowledge to health care professionals [8]. Proponents of the use of social media for knowledge translation in health care point to three key features that make these tools highly effective: personalization, presentation, and participation [9]. The tailoring of content allows users to access and share information that is most valuable to them, whereas the versatility of social media creates numerous options for the presentation of information. The immediacy of social media also facilitates timely information sharing, and the availability of multiple formats (eg, blogging platforms, microblogging sites, and social networking sites) allows for flexible dissemination options, depending on the purpose of the tools and the preferences of the target population. The collaborative nature of social media allows for a meaningful contribution from all user groups [5]. Finally, social media can incorporate components of traditional knowledge translation interventions that have demonstrated effectiveness in changing health care providers’ behavior, including the combination of didactic and interactive content in the distribution of educational materials [10] and endorsement by local opinion leaders [11].

The body of literature exploring social media and their utility in health care is rapidly growing; however, focus is primarily given to social media as a tool that patients can utilize to support their health and how social media can be used to enhance communication between patients and health care professionals [1]. A recent integrative review reported a modest level of evidence that a desire to gain and exchange knowledge is a primary motivator for social media use by health care professionals [12]. The evidence suggests that clinicians communicate via social media mostly within their discipline and that gaining access to new knowledge is an essential benefit of engagement with social networks and virtual communities [12]. Despite the potential for social media as a knowledge translation strategy in health care and the enthusiasm surrounding its use, there is a lack of empirical and longitudinal studies examining the effectiveness of using social media as a basis for a knowledge mobilization strategy aimed at health care professionals [13].

Cochrane (formerly known as the Cochrane Collaboration) is an international network of health researchers, professionals, and consumers who work together to synthesize high-quality, trusted evidence to enhance health care knowledge and decision making [14]. The members of Cochrane translate review evidence for different audiences using a variety of formats such as decision aids, plain language summaries of Cochrane systematic reviews, and podcasts [15]. Cochrane entities, including Cochrane Musculoskeletal, Cochrane Croatia, and Cochrane Schizophrenia, have experimented with the use of social media tools (Facebook and Twitter) to disseminate Cochrane summaries [15-17]. Between March 2013 and June 2014, Cochrane Croatia measured Facebook activity for a page sharing Croatian translations of Cochrane summaries and gained 1441 followers. Contributing to the empirical evidence of the effectiveness of social media dissemination, Cochrane Schizophrenia designed a randomized controlled trial to assess the impact of tweeting Cochrane summaries and showed a nearly threefold increase in Web visits to the tweeted Cochrane content [17].

Cochrane Child Health advocates for and facilitates the conduct of systematic reviews on child health topics [18]. To build on the exploratory use of social media for knowledge translation of Cochrane evidence, the objectives of this study were to implement and evaluate a structured social media strategy using multiple platforms to disseminate child health–relevant Cochrane systematic reviews and summaries to health care providers caring for children.

## Methods

### Summary of Promotional Activities

For a 22-week study period between November 3, 2014 and April 5, 2015, we promoted high-quality child health evidence to professionals providing health services to children and youth through Cochrane Child Health’s social media presences. Our strategy comprised three key components: (1) a weekly blog post, (2) daily messages on Twitter (“tweets”), and (3) a monthly journal club hosted on Twitter as a “tweet chat.” We collected data on Web traffic and user engagement through metrics provided by a series of Web-based analytics tools described below.

### Identification of Evidence and Vetting of Content

Our selection of evidence to promote was guided by content contained in child health–relevant systematic reviews within the Cochrane Database of Systematic Reviews. Our efforts were informed by popular and topical Cochrane content, material included in *Evidence-Based Child Health: A Cochrane Review Journal* (eg, overviews of reviews and podcasts), and collaborations with other bloggers associated with Cochrane. Each week, we chose a new topic to profile as the focus of our blog posts and tweets. Our goal was to represent high-quality evidence across a spectrum of child-relevant issues to appeal to a diverse group of child health care providers. The blog posts were drafted by study investigators (MPD, ASN, DT, and LH), study staff (KS and AW), or content experts. All content (ie, blog posts and tweets) was reviewed by study investigators, and relevant content experts as needed, to ensure the accuracy of information shared.

### Social Media Strategy

Our strategy focused on two commonly used social media tools: blogs and Twitter [19]. At the beginning of every week during the study period, we added a new post to our WordPress blog, summarizing the key messages from the Cochrane review or overview being featured that week (see Multimedia Appendix 2 for blog titles and topic categories). The blog posts were written with the goal of being succinct and written in plain language to facilitate uptake by our end users [20]. We also incorporated images and maximized the use of white space to increase visual appeal [21]. Along with our summary, we provided an appraisal of the evidence and links to the original research and supplementary material such as podcasts, Cochrane plain language summaries, and patient resources (see Multimedia Appendix 3 for a sample blog post).

For the remainder of each new week, we promoted our blog post and the evidence on Twitter (@Cochrane_Child). We published 21 tweets per week (3 tweets per day, 7 days per week). We used the Web-based scheduling tool Twuffer to ease daily resource demands and timed our tweets to be released in the morning, afternoon, and evening (local Mountain Standard Time). Common Twitter hashtags (keywords or phrases preceded by a hash symbol [#] to identify messages on specific topics) and handles (Twitter account names preceded by the @ symbol) for relevant pediatric interest groups were included in every tweet. The @Cochrane_Child Twitter account was monitored by a research staff member to respond to comments and engage with followers as applicable.

Over the course of the 22-week period, we hosted 3 journal clubs (tweet chats) on Twitter (see Multimedia Appendix 4 for journal club titles and topic categories). Each tweet chat was an hour long and took place at a prescheduled date and time. We promoted the meetings with blog posts and tweets. For our tweet chat meetings, we also recruited clinical experts known to the research team on the chosen topics to participate. Our expert collaborators helped us identify key discussion points related to the quality and applicability of the evidence. We used these discussion points to help facilitate the tweet chat and also to promote the event. The tweet chats were intended to be informal, allowing for discussion among and questions from participants. However, we prepared tweets and a rough script for the meeting to maintain a consistent format and to ensure that all key points would be addressed. Journal club meetings were recorded and archived using the Web-based tool Storify. We promoted the archived tweet chats on the WordPress blog and Twitter.

### Evaluation

#### Blog

Web traffic to and user engagement with the blog were measured using the built-in analytics program in WordPress. We tracked the numbers of page views and visitors per day, sources of site referrals, and the geographic spread of our visitors. Blog posts were open for comments, which we collected and analyzed.

#### Twitter

Twitter analytics and Twitonomy were used to obtain detailed metrics for our tweets, including engagement (number of times users interacted with our tweets), impressions (potential number of times users viewed our tweets), and the number of retweets (number of times users shared our tweets with their followers), clicks, favorites, and followers. We tracked specific tweets that received the most attention and classified these using the Cochrane review group structure to clarify the relevant clinical area. We also collected data on the number of followers the @Cochrane_Child Twitter account gained during the promotion and their behavior over the study period. Where possible, we extracted descriptive data (eg, profession, affiliation, and self-reported interests) on our followers from their public profiles.

#### Journal Clubs

Following each journal club, we asked participants to complete a brief survey using Google Forms. The survey was distributed via Twitter and comprised 8 multiple-choice and free-text questions (available in Multimedia Appendix 5). Ethics approval for the survey was obtained from the Health Research Ethics Board at the University of Alberta. For the tweet chat journal clubs, we also recorded the number of views each of the archived meetings received on Storify.

#### Accessing the Original Publications

To evaluate the impact of our social media strategy on the frequency that the original publications were accessed, we used traceable links generated by Bitly and alternative social media metrics (altmetrics) through Altmetric.com scores. For all hyperlinks that we posted on our blog and Twitter, we used shortened, unique Web addresses (URLs) created by Bitly. The Bitly account collected data on the number of clicks each link received, allowing us to directly measure audience interaction with our social media posts. Altmetrics measure Web-based attention based on how far scholarly content travels through the social Web and encompass reflections of both the quantity and the quality of attention received [22]. The Altmetric.com score is an automatically calculated weighted count of all attention a publication has received, based on the volume, sources, and authors [23]. The score increases as more people mention the publication; however, the amount by which the score will increase varies based on the source of the mention (eg, the score will increase more if the source of the mention was a newspaper vs a tweet), self-promotion by authors, potential bias toward a journal or a publisher, and whether the content is being shared directly with its intended audience [23]. A decrease in the score may occur in unusual cases because of a fluctuation. A fluctuation can result from a tweet being removed by the original tweeter, a Twitter account being deemed “biased” according to an Altmetric.com moderator, or because of a change made to the algorithm being used to calculate the score [24]. For each publication we shared throughout the study, we collected Altmetric.com data before, during, and after the promotion effort. We attempted to collect data on article downloads for the promoted Cochrane systematic reviews from the publisher, but only the annual numbers of downloads and page views were available. We were unable to obtain information specific to our study period.

#### Resource Implications

Using an internal time log, we tracked data on the staff time dedicated to this project, including the upkeep of the social media accounts, identifying content, writing and publishing blog posts and tweets, and participating in journal club meetings. The data available do not reflect the time committed by the study investigators and other content experts.

### Analysis

We used descriptive statistics, including numbers, frequencies, and means, to analyze quantitative data and content analysis to analyze qualitative data where applicable [25]. We collected data on our blog site, Twitter account, and Altmetric.com scores at the outset to establish a baseline of usage to compare data collected during the promotion; we continued to collect data for click counts and page views for 1 month beyond the promotion to allow our messages and posts time to be viewed and shared on social media sites. Popularity of blogs was determined by the number of visitors and views, and popularity of tweets was determined by the number of retweets, favorites, engagements, impressions, and URL click counts. The results were graphically displayed.

## Results

### Summary of Web-Based Attention Received

**Figure 1:**
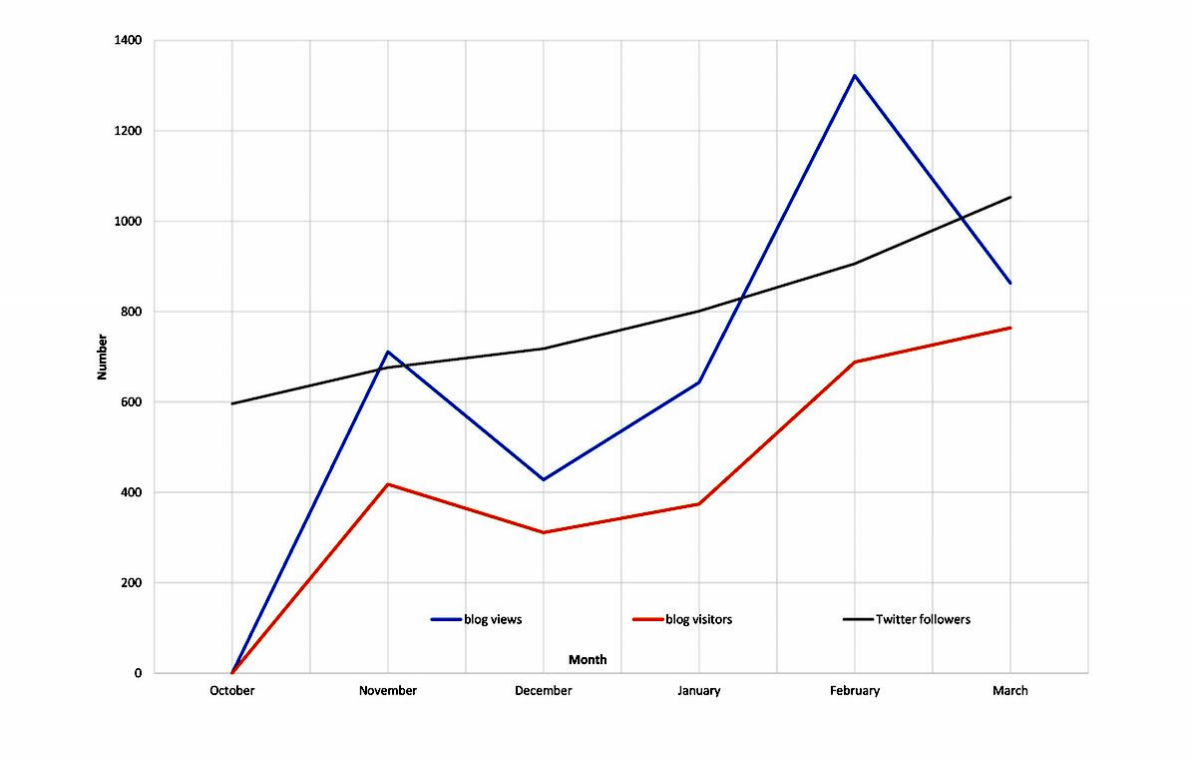
Engagement with the Cochrane Child Health blog and @Cochrane_Child Twitter account over time.

Over the study period, we shared 25 blog posts (22 topic posts and 3 journal club announcements; Multimedia Appendices 2 and 3), published 585 tweets (434 promoting the blog posts, 36 promoting the journal clubs, and 115 during journal club meetings), and hosted 3 journal club sessions. Overall, the blog received a total of 2555 visitors and 3967 page views, and the Twitter account gained 469 new followers from a baseline of 596 followers to 1065 followers at study completion (representing a 79% increase, 469/5.96). The degree of monthly engagement with both the blog and Twitter account increased over time (Figure 1).

### Web Traffic and User Engagement

The following data were collected during the period October 6, 2014 to April 24, 2015, and data collection overlaps the study period by approximately 1 month on either end. Most views of the blog (50%, 1996/39.67) originated via referrals from Twitter, followed by Facebook (16%, 640/39.67). Facebook referrals were likely mainly from the accounts managed by Cochrane and Cochrane Canada. Also, one of our research collaborators (RF) had linked Facebook and Twitter accounts, which mirrored posts composed on each site. Of the total views, 1.4% (56/39.67) (n=56) originated from the Cochrane Child Health website. Geographically, views were mainly from Canada (29%, 1181/39.67) and the United Kingdom (28%, 1120/39.67); however, the remaining views were distributed among an international audience (North America, excluding Canada: 24%, 946/39.67; Europe: 23%, 897/39.67; Australia: 5%, 207/39.67; Africa: 3%, 117/39.67; South America: 4%, 175/39.67; and Asia: 1%, 44/39.67).

The most popular blog topics were (1) “Honey: An effective cough remedy for kids?” (n=241 visitors; n=359 page views); (2) “To wheeze or not to wheeze” (long-acting beta2-agonists [LABAs] for asthma) (n=216 visitors; n=340 page views); and (3) “Lactose avoidance: Worthwhile for reducing duration of diarrhea in kids?” (n=215 visitors; n=304 page views). These 3 blog posts were published in consecutive weeks (study weeks 16-18), and “LABAs for asthma” was the topic of our second journal club (during study week 17). Overall, 3 posts generated comments from viewers: “The power of touch: Skin-to-skin contact and kangaroo mother care for newborns” (link to evidence regarding skin-to-skin contact and pain management), “To wheeze or not to wheeze” (clinical comment regarding use of LABAs and inhaled corticosteroids for asthma), and “Children and youth with obesity—A growing global epidemic” (notification that our post had been reblogged).

Our Twitter account attracted mainly individuals who identified as health care providers (n=206) and academics (n=107). We also gained some attention from individuals representing organizations such as hospitals, nonprofit organizations, and professional associations (n=65). Self-reported interests often included child health and care (n=120), with additional categories including health evidence and resources (eg, evidence-based practice and knowledge translation; n=29) and social justice, advocacy, and policy (n=27). The classifications reported were not mutually exclusive.

Across clinical topics, our tweets were most frequently in the areas of acute respiratory infections (ARI; n=139), neonatology (n=43), and airways (n=42). Twitter followers were highly engaged with tweets related to public health (mean 4.5 retweets per tweet and 1.7 favorites per tweet) and pain (mean 3.6 retweets per tweet and 1.6 favorites per tweet). These results are summarized in Table 1. For individual tweets, the highest numbers of retweets were in the areas of ARI (antivirals for influenza; n=17), public health (obesity prevention programs; n=13), and airways and ARI (promotion for asthma journal club and measles-mumps-rubella [MMR] vaccination; n=12 each). Impressions were highest for 2 tweets about the MMR vaccine (n=3151 and 2983) and public health (n=2374), engagement was greatest for a tweet related to sucrose/glucose for infant pain (n=51), and the largest number of URL clicks were in tweets related to ARI (antibiotics for sore throat, n=28; bronchiolitis, n=20) and neonatology (procedural pain, n=25). See Table 2 for details.

**Table 1:** Summary of Twitter activity by tweet topic.

Topic	Total tweets (N=4.34), n (%)	Total retweets (N=10.28), n (%)	Mean retweets per tweet	Total favorites (N=3.57), n (%)	Mean favorites per tweet
Acute respiratory infections	139 (32)	318 (31)	2.3	126 (35)	0.91
Neonatology	43 (10)	92 (9)	2.1	23 (6)	0.53
Airways	42 (10)	80 (8)	1.9	47 (13)	1.1
Anxiety, depression, and neurosis	22 (5)	43 (4)	2	11 (3)	0.5
Cystic fibrosis and genetic disorders	22 (5)	19 (2)	0.86	4 (1)	0.18
Fertility regulation	21 (4.8)	36 (4)	1.7	9 (3)	0.43
Infectious diseases	21 (4.8)	45 (4)	2.1	16 (5)	0.76
Injuries	21 (4.8)	35 (3)	1.7	4 (1)	0.19
Pain, palliative and supportive care	21 (4.8)	76 (7)	3.6	34 (10)	1.6
Psychosocial and learning problems	21 (4.8)	43 (4)	2	8 (2)	0.38
Skin disorders	21 (4.8)	18 (2)	0.86	12 (3)	0.57
Cancer	20 (4.6)	134 (13)	3.4	29 (8)	0.73
Public health	20 (4.6)	89 (9)	4.5	34 (10)	1.7
Total	434	1028	2.23	357	0.74

**Table 2:** Summary and ranking of four most popular tweets by specific metrics.

Tweet topic	Tweet content	Retweets, n (rank)	Favorites, n (rank)	Impressions^a^, n (rank)	Engagement^b^, n (rank)	URL clicks, n (rank)
Acute respiratory infections	Do antivirals work for #flu? @cochranecollab evidence concludes they’re not as effective as we previously thought	17 (#1)	3 (-)^c^	2175 (#4)	50 (#2)	7 (-)
Public health	#systematicreview of 37 studies w/27,964 children shows #obesity prevention programs reduce adiposity	13 (#2)	6 (#3)	1400 (-)	44 (-)	12 (-)
Journal club promotion	Passionate about #childhealth evidence? Join the #CochraneChild tweet chat Feb 25 @ 2 pm MST (9 pm GMT)	12 (#3)	6 (#2)	1974 (-)	45 (-)	8 (-)
Acute respiratory infections	Measles is making a comeback | #itsasmallworldafterall, so get vaccinated!	12 (#3)	1 (-)	3151 (#1)	32 (-)	3 (-)
Pain, palliative and supportive care	Pain relief important part of caring for kids | we need to address knowing-doing gap. #painevidence	11 (#4)	4 (-)	1409 (-)	45 (-)	13 (-)
Neonatology	No more research needed on sucrose/glucose for infants pain | Now we need to put knowledge into action!	11 (#4)	5 (#4)	1690	51 (#1)	25 (#2)
Acute respiratory infections	Blogging #childhealth evidence | This week: Cochrane meets controversy: Vaccines for measles, mumps, & rubella	11 (#4)	4 (-)	2983 (#2)	43 (-)	13 (-)
Public health	Blogging #childhealth evidence | This week: Policies & strategies for preventing childhood #obesity	11 (#4)	5 (#4)	2347 (#3)	39 (-)	6 (-)
Acute respiratory infections	Coughing kids? #honey better than placebo or diphenhydramine for improving sleep in children (and parents)	10 (-)	6 (#1)	1149 (-)	46 (-)	12 (-)
Acute respiratory infections	1 out of 3 babies will get #bronchiolitis in their 1st year | Despite its prevalence, clinical practice varies	7 (-)	3 (-)	823 (-)	49 (#3)	20 (#3)
Infectious diseases	Oral rehydration? Lactose avoidance? Both? | @giordanopg talks about how to treat acute diarrhea in kids	7 (-)	5 (-)	1186 (-)	49 (#3)	15 (-)
Acute respiratory infections	Are antibiotics over-prescribed for sore throat? @UKCochraneCentr | #childhealth evidence	6 (-)	5 (-)	1525 (-)	47 (#4)	28 (#1)

^a^Impressions reflect the number of times a user is served a tweet in timelines or search results.

^b^Engagement reflects the total number of times a user interacted with a tweet.

^c^(-) indicates that the tweet did not rank in the top four.

Tweet chats had limited numbers of active Web-based participants but gained usage through a Web-based repository where the journal clubs could be accessed after the meeting. The archived tweet chats on Storify received between 37 and 57 views each: January—bronchiolitis (n=57); February— asthma (n=22); and March—obesity prevention (n=37). Three participants responded to the surveys administered after each journal club: one in response to the bronchiolitis journal club and two in response to the asthma journal club. All 3 participants were physicians, with 1 physician being interested in Twitter generally and the other 2 physicians being specifically interested in the content (asthma). The perceived benefits of hosting a journal club on Twitter included it being a useful tool to check understanding of the subject matter and gain new ideas, as well as providing a level playing field for everyone interested in participating. A suggestion to improve the format of the journal club was to include key images with the tweets to add to the discussion.

### Accessing the Original Publications

Over the course of the study, we created 183 customized, traceable Bitly links, which received 3463 clicks. Just over half of these clicks (55%, 1892/34.63) were directed to the Cochrane Child Health blog home page. As our new posts always appeared on the blog’s home page, we consistently promoted the home page link on social media channels. A considerable proportion of clicks (14%, 468/34.63) were related to the journal clubs, including the studies being discussed (7%, 232/34.63), links to the announcements (4%, 141/34.63), and archived discussions (2%, 61/34.63). The Cochrane review on interventions for preventing obesity in children was the most highly accessed (n=93 clicks; see Table 3; [26]). Other commonly accessed studies included those on oral antihistamine-decongestant- analgesic combinations for the common cold (n=99) and procedural pain in children (n=59; [27,28]).

We collected Altmetric.com data for 61 studies promoted during the study period (Multimedia Appendix 6). The mean change in score was an increase of 11 points (median: 5; range: −1 to 73). Usage for our study corresponded to an average of 10 clicks on the Bitly links to the studies that we promoted (median: 3; range: 0-97) and a mean change in Altmetric.com score per Bitly click of 3 (median: 1.5; range: −0.25 to 37). Most attention for these studies came from Twitter (n=2229 tweeters), Mendeley (n=981 readers), and Facebook (n=400 timelines). The topics of studies that experienced the greatest Altmetric.com score increase included the following: (1) neuraminidase inhibitors for preventing and treating influenza, (2) vitamin C for the common cold, and (3) zinc for the common cold (see Table 4). Of our journal club studies, the Altmetric.com scores increased by 36 points (range: 104-139) for the review on interventions for preventing obesity for children [26], 17 points (range: 0-17) for the review on LABAs for asthma [29], and 11 points (range: 0-11) for the overview on the treatment of bronchiolitis [30]. These scores placed the reviews and overviews in the 99th, 95th, and 88th percentiles, respectively, for Altmetric.com scores of studies of the same age and published in any journal.

**Table 3:** Most commonly accessed links.

Rank	Resource title link	Link type	Clicks
#1	Cochrane Child Health blog home page	Blog home page	1892
#2	Oral antihistamine-decongestant-analgesic combinations for the common cold	Cochrane systematic reviews	99
#3	The Cochrane Library and procedural pain in children: An overview of reviews	Cochrane overview of systematic reviews	97
#4	Interventions for preventing obesity in children	Cochrane systematic reviews	93
#5	The Cochrane Library and the treatment of bronchiolitis in children: An overview of reviews	Cochrane overview of systematic reviews	77
#6	Journal club announcement: LABAs for asthma	Journal club announcement	65
#7	The Cochrane Library and safety of regular long-acting beta2-agonists in children with asthma: An overview of reviews	Cochrane overview of systematic reviews	62
#8	The Cochrane Library and procedural pain in children: An overview of reviews [podcast]	Podcast	59
#9	Journal club announcement: Evidence for treatment of bronchiolitis	Journal club announcement	38
#9	Journal club announcement: Obesity prevention	Journal club announcement	38
#10	The Cochrane Library and the treatment of sore throat in children and adolescents: An overview of reviews	Cochrane overview of systematic reviews	37
#10	Does this patient have strep throat? The rational clinical examination	External evidence-based medicine resource	37
#10	Honey for acute cough in children	Cochrane systematic reviews	37

**Table 4:** Top Altmetric.com score growth among promoted studies.

Article title	Journal	Total score increase, points (% increase)	Baseline score	Final score
Neuraminidase inhibitors for preventing and treating influenza in healthy adults and children	Cochrane Database of Systematic Reviews (CDSR)	73 (23)	312	385
Vitamin C for preventing and treating the common cold	CDSR	53 (18)	295	348
Zinc for the common cold	CDSR	40 (15)	271	311
Interventions for preventing obesity in children	CDSR	36 (35)	104	140
Honey for acute cough in children	CDSR	35 (97)	36	71

### Resource Implications

Not including the time invested by the study investigators to write and review blog posts and tweets and to plan and participate in the journal club meetings, research staff (n=3) contributed approximately 433 hours to the project (approximately 6.5 hours each per study week). We involved a research librarian, a project coordinator, and a graduate student throughout the project; 2 undergraduate summer students were also briefly involved. These members of the team led the coordination of our Twitter activity and logistics of managing the blog and also contributed to drafting blog posts.

## Discussion

### Empirical Evidence of Social Media for Knowledge Dissemination

This study provides empirical evidence on the potential impact of social media activities for knowledge translation in the health sciences. We implemented a structured 22-week social media strategy involving a coordinated approach using commonly accessed social media platforms (ie, WordPress blog and Twitter) and demonstrated that engagement with our blog and Twitter account increased steadily over time and was geographically diverse. However, we found our approach was resource intensive and required the involvement of several content experts.

Social media platforms have been widely explored in the context of facilitating communication and improving knowledge among health care professionals [4]. Our findings demonstrate that a coordinated social media strategy may be an effective approach for sharing health evidence among a geographically diverse audience of health care providers, academics, and health care organizations. Although our promotional activities originated in Canada, we also attracted attention across North America, Europe, Australia, Africa, Asia, and South America. Similarly, in a study conducted by Cochrane Croatia utilizing Facebook as a dissemination tool for Cochrane summaries, the intended audience was initially nationally focused but ultimately grew to include followers worldwide [16]. Ultimately, the reach of social media can far surpass intended immediate audiences, which reinforces the potential utility of social media tools for extending the global reach of health research.

Although social media can be an effective means for broadly sharing health research, the process is resource intensive and requires careful planning. To realize our social media strategy, the research staff (n=3) contributed approximately 433 hours to promotion activities and planning (6.5 hours each per week). In addition to research staff hours, each week, the study investigators were involved in reviewing all content (blog posts and tweets). Content experts provided a quote on the relevant review and, for journal clubs, moderated discussion for 1 hour. Investigators and content experts were involved to ensure information accuracy; however, this process was resource intensive and involved a trade-off in terms of effort for yield. Moreover, engaging content experts can be challenging because of the time required without formal acknowledgment of their contributions (eg, through academic recognition).

### Strengths and Limitations

Previous research on disseminating Cochrane evidence through social media has primarily utilized single platforms such as Facebook [16] or Twitter [17] and has minimally utilized Web analytics (eg, page views or number of followers) to provide a quantitative impact assessment. To the best of our knowledge, this is the first study where a Cochrane group or knowledge translation program has evaluated a social media strategy using coordinated platforms (eg, Twitter and WordPress blogging) and a range of analytics (altmetrics, click counts, page views, site visits, and engagements) from different sources (Altmetric.com, Twitter analytics, Bitly, and WordPress analytics) to create a more complete and quantifiable usage picture. Recently, Cochrane review groups have highlighted their support for journal club activities [15], yet little research describing and evaluating the process of conducting journal clubs via Twitter (tweet chats) has been conducted. Our social media strategy provides insight with regard to this gap in the published literature through the development, implementation, and quantitative assessment of 3 tweet chats. Additionally, the quantification of required resources and staff time is an additional contribution of this study to the literature on social media for knowledge dissemination and will allow other research teams to estimate the time from staff and others needed to undertake such a project. This information is useful to a range of users, including those designing social media strategies, those interested in disseminating health information in general, and those interested in promoting specific items (eg, authors or publishers wishing to promote specific items).

Limitations of this study include a variable range of reporting periods for the statistics. Some statistics from free Web-based tools (ie, Bitly) were only available for a limited period of 30 days. In the case of Bitly click counts on our traceable URLs, we recorded data from the last 30 days of the promotion. In other cases, available statistics covered a longer period (ie, WordPress) than the 22-week promotion. WordPress blog site statistics were available for “all time” and extended back to when the blog was first created on October 6, 2014. The extended period of data collection may have contributed to an overestimate to usage than what we can directly attribute to our promotion. However, as the blog had very little activity before the promotion started, we do not consider that this extended reporting period contributed to a significant increase in usage. In the case of the journal clubs, we were unable to measure the number of passive Twitter users who viewed the tweet chat during the events. Recorded statistics for the Web archive of the journal clubs extended long past the time of the meetings (recorded in December 2016). Although inconsistent with the rest of our data collection activities, the tweet chat archive suggests continuing use of the materials, and we felt this information was worth collecting. A further limitation of the freely available analytics tools was an inability to exclude statistics from internal use. Our project team was aware of this problem and made every effort to limit the amount of site testing and internal clicks on our traceable links. There is, however, a possibility that some of our recorded numbers represent usage from our own team. Our study was also limited by the method in which Altmetric.com scores are informed. Not only do the Altmetric.com data capture our influence on evidence uptake, but they also encompass the impact of other sources during the same time frame. Due to this, we cannot distinguish between the impact of our social media promotion on article Altmetric.com scores and that of other sources. Finally, a greater potential challenge of this study, and to all dissemination research related to health evidence, is our inability to attribute knowledge sharing to behavior change and improvement in health outcomes. Our use of proxy measures allows for an overview of potential use of evidence; however, future research is needed to determine the health impact of knowledge-sharing activities, as well as methods to gather this information.

### Lessons Learned and Future Directions

We have identified several lessons learned (see Textbox 1) from our study that we believe are applicable to individuals interested in utilizing social media as a knowledge dissemination strategy across a variety of academic and professional fields. Foremost, the awareness of the amount of time and effort required to manage the social media strategy, including identifying and creating content, is paramount. In this study, our recorded time was limited to what was invested by research staff; investigators and content experts (eg, clinicians and guest bloggers) invested additional time weekly that was not captured in our results. Also important to consider are the specific objectives or purposes of the social media strategy. We focused our efforts almost entirely on dissemination and thus generated very minimal interactivity, which in many cases is one of the intended advantages of social media. Further research evaluating processes and outputs related to purposeful approaches to increased interactivity is required. However, increasing interactivity will require greater investment in terms of resources and time. This, in part, may be mediated by pursuing focused topic areas and utilizing existing established networks. Finally, considering the scope of topics included in the social media strategy and the associated resource implications of the chosen scope would be of benefit. In this study, we found our broad focus (ie, child health evidence) and our approach of changing specific topics each week to be challenging in terms of finding and engaging numerous content experts. Social media strategies within specific content areas may be more effective at developing a social network or engaging within an existing Web-based community, as well as activating relevant opinion leaders. Subsequently, a more content-specific approach may have a greater impact on information uptake in practice.

Priorities for future research include identifying metrics to assess the impact of social media and a given social media strategy’s intended effects. Furthermore, conducting research focusing on a better understanding of the experiences and motivations of social media users and the uptake of disseminated information in practice would be of benefit.

**Lessons learned.**

- Consider allocation of time and budgeting resources for all personnel before commencing the social media strategy
- Consider the specific objectives of the social media strategy
- Consider the scope of topics to be covered in the social media strategy (eg, narrow or broad)
- Utilize existing established networks when available and feasible

### Conclusions

Our coordinated knowledge dissemination strategy allowed us to gather empirical evidence on how social media can be used to share research evidence with an audience of child health professionals. We increased Web-based followers for our social media presences and, using proxy measures, observed an increase in access to the evidence we were promoting. Time tracked by our team members provides an estimate for researchers planning to undertake a similar promotion using social media tools. The range of analytics included in this study contributes to our understanding of how to assess the reach and impact of knowledge dissemination activities via social media. Our methods of using coordinated activities via multiple social media platforms expand on existing dissemination practice and explore numerous opportunities to enhance health research promotion.

## Appendix

Multimedia Appendix 1 Glossary of social media terms.

Multimedia Appendix 2 WordPress blog post topics by week.

Multimedia Appendix 3 A sample post from the Cochrane Child Health WordPress blog.

Multimedia Appendix 4 Journal club topics by week.

Multimedia Appendix 5 Journal club survey questions.

Multimedia Appendix 6 Altmetric.com scores for all promoted articles.

## Abbreviations

altmetrics: alternative social media metrics
ARI: acute respiratory infection
LABA: long-acting beta2-agonist
MMR: Measles-Mumps-Rubella
