# Supplementary material for: Social Media for the Dissemination of Cochrane Child Health Evidence: Evaluation Study
Source: J Med Internet Res. 2017 Sep 1;19(9):e308. doi: 10.2196/jmir.7819 (PMC5600964; doi:10.2196/jmir.7819)
Supplement: Multimedia Appendix 1 [file jmir_v19i9e308_app1.pdf]

Appendix F. Glossary of Social Media Terms

bit.ly – An online service for generating short, traceable web addresses (URLs)

blog post – A discreet article, column or publication distributed by an online publishing platform (e.g., WordPress)

favorite – A Twitter action to indicate your interest in a message

follower – A Twitter account subscribed to receive messages from your account

handle – A Twitter short name for an account. Handles are used to reply to or share tweets. Twitter handles are prefixed by the @ symbol (e.g., @Cochrane_Child)

hashtag – A hashtag describes the subject of a tweet and is included in the message. Users may track particular hashtags to participate in online tweet chats or to follow topics of interest (e.g., #ChildHealth)

retweet – A Twitter action to share a message with your followers

tweet – A message on Twitter

tweet chat – An synchronized meeting or discussion on Twitter. Users participate by using the same hashtag in their tweets

Twitter – A micro-blogging site where short messages (tweets) of 140 characters or less are distributed by an account

Twuffer – An online tool to compose and schedule tweets

WordPress – A blogging platform that facilitates online publishing
