# Supplementary material for: Social Media for the Dissemination of Cochrane Child Health Evidence: Evaluation Study
Source: J Med Internet Res. 2017 Sep 1;19(9):e308. doi: 10.2196/jmir.7819 (PMC5600964; doi:10.2196/jmir.7819)
Supplement: Multimedia Appendix 2 [file jmir_v19i9e308_app2.pdf]

Appendix A. WordPress blog post topics by week

Week	Blog Post Title	Blog Post Topic[a]
1	Making medical procedures less painful for kids – the evidence	Pain, Palliative and Supportive Care
2	Kids and asthma: Should we worry about growth?	Airways
3	How can we do this better?	Neonatology; Pain, Palliative and Supportive Care
4	Treating the #1 mental disorder in kids and teens	Anxiety, Depression and Neurosis
5	A normal day at school… Cystic fibrosis and me	Cystic Fibrosis and Genetic Disorders
6	The power of touch: Skin-to-skin contact and kangaroo mother care for newborns	Neonatology, Pregnancy and Childbirth
7	Will over-the-counter cough and cold medications help our kids feel better this cold season?	Acute Respiratory Infections
8	Do vitamin C and zinc work for the common cold?	Acute Respiratory Infections
9	Evidence for eczema prevention falls short – management is key	Skin Disorders
10	Scared straight: Scary evidence	Psychosocial and Learning Problems
11	Too good to be true? The case of influenza antivirals	Acute Respiratory Infections
12	Interventions for the treatment of bronchiolitis in infants	Acute Respiratory Infections
13	Evidence for the treatment of sore throat in children and teens	Acute Respiratory Infections
14	Graduated driver licensing decreases motor vehicle crashes – the evidence and the uptake	Injuries
15	The evidence behind preventing unintended pregnancies in adolescents	Fertility Regulation
16	Honey: An effective cough remedy for kids?	Acute Respiratory Infections
17	To wheeze or not to wheeze?	Airways
18	Lactose avoidance: Worthwhile for reducing duration of diarrhea in kids?	Infectious Diseases
19	Cochrane meets controversy: Vaccines for measles, mumps, and rubella	Acute Respiratory Infections
20	Treating children with cancer: Looking to the future	Childhood Cancer
21	Children and youth with obesity – a growing global epidemic	Public Health
22	Communicating with young people in hospital: How can we get it right?	Childhood Cancer; Consumers and Communication

[a]Clinical area illustrated by Cochrane review group
