# Supplementary material for: Social Media for the Dissemination of Cochrane Child Health Evidence: Evaluation Study
Source: J Med Internet Res. 2017 Sep 1;19(9):e308. doi: 10.2196/jmir.7819 (PMC5600964; doi:10.2196/jmir.7819)
Supplement: Multimedia Appendix 3 [file jmir_v19i9e308_app3.pdf]

Appendix B. A sample post from the Cochrane Child Health WordPress blog

Lactose avoidance: Worthwhile for reducing duration of diarrhoea in kids?

1

Posted on **March 2, 2015**

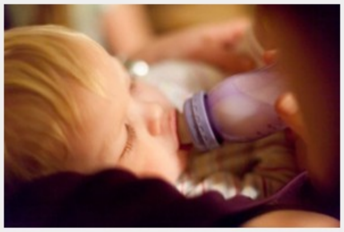

— Source: Wikimedia Commons

One of the great advances in medicine in the 20th century was the introduction of oral rehydration therapy. Thanks to this simple, affordable therapy, mortality from acute diarrhoea has all but disappeared in middle and high income countries. It also has reduced mortality in low income countries but there is still much work to do. This work includes the World Health Organisation's plan to end preventable child deaths from diarrhoea by 2025 (WHO 2013). While this is a first priority in the treatment of children with diarrhoea, another important step is to reduce the burden of disease.

Acute diarrhoea is commonly caused by infectious gastroenteritis. The most frequent cause remains rotavirus infections (although, thanks to the vaccine, it is also decreasing). And since some infections result in transient lactose intolerance it sounds logical to avoid lactose for a while.

Logical, yes. But is it practical?
