# Supplementary material for: Social Media for the Dissemination of Cochrane Child Health Evidence: Evaluation Study
Source: J Med Internet Res. 2017 Sep 1;19(9):e308. doi: 10.2196/jmir.7819 (PMC5600964; doi:10.2196/jmir.7819)
Supplement: Multimedia Appendix 4 [file jmir_v19i9e308_app4.pdf]

Appendix C. Journal club topics by week

Week	Journal Club Post Title	Journal Club Topic[a]
12	Evidence for treatment of bronchiolitis | Cochrane Child Health Twitter Journal Club - #CochraneChild	Acute Respiratory Infections
17	LABAs for asthma | Cochrane Child Health Twitter Journal Club - #CochraneChild	Airways
21	Obesity prevention | Cochrane Child Health Twitter Journal Club - #CochraneChild	Public Health

[a]Clinical area illustrated by Cochrane review group
