# Supplementary material for: Social Media for the Dissemination of Cochrane Child Health Evidence: Evaluation Study
Source: J Med Internet Res. 2017 Sep 1;19(9):e308. doi: 10.2196/jmir.7819 (PMC5600964; doi:10.2196/jmir.7819)
Supplement: Multimedia Appendix 5 [file jmir_v19i9e308_app5.pdf]

Appendix D. Journal club survey questions

1) Did you learn something new from the journal club?
2) How will you use the information you received in the journal club?
3) How did you participate in the discussion?
4) Would you attend another journal club hosted by the Cochrane Child Health Field?
5) Would you recommend the Cochrane Child Health Twitter journal club?
6) Please suggest child health topics for future journal clubs
7) How can we improve the format of the journal club?
8) What is your primary professional role?
