# Supplementary material for: Social Media for the Dissemination of Cochrane Child Health Evidence: Evaluation Study
Source: J Med Internet Res. 2017 Sep 1;19(9):e308. doi: 10.2196/jmir.7819 (PMC5600964; doi:10.2196/jmir.7819)
Supplement: Multimedia Appendix 6 [file jmir_v19i9e308_app6.pdf]

Appendix E. Altmetric.com scores for all promoted articles

**Title**	**Journal**	**Baseline (before promotion)**	**Score after promotion**	**Change from Baseline**
Optimal duration of exclusive breastfeeding	Cochrane database of systematic reviews	54	69	15
Behavioural and cognitive behavioural therapy for obsessive compulsive disorder in children and adolescents.	Cochrane database of systematic reviews	0	5	5
Topical pimecrolimus for eczema	Cochrane database of systematic reviews	1	6	5
Probiotics for treating eczema.	Cochrane database of systematic reviews	9	13	4
Dietary supplements for established atopic eczema.	Cochrane database of systematic reviews	17	20	3
Oral evening primrose oil and borage oil for eczema	Cochrane database of systematic reviews	87	90	3
Pharmacotherapy for anxiety disorders in children and adolescents.	Cochrane database of systematic reviews	0	3	3
Soy formula for prevention of allergy and food intolerance in infants.	Cochrane database of systematic reviews	0	3	3
Formulas containing hydrolysed protein for prevention of allergy and food intolerance in infants	Cochrane database of systematic reviews	3	5	2
Maternal dietary antigen avoidance during pregnancy or lactation, or both, for preventing or treating atopic disease in the child	Cochrane database of systematic reviews	8	10	2
Cognitive behavioural therapy for anxiety disorders in children and adolescents.	Cochrane database of systematic reviews	32	32	0
Omega 3 and 6 oils for primary prevention of allergic disease: systematic review and meta-analysis.	Allergy	1	1	0
Prebiotics in infants for prevention of allergy.	Cochrane database of systematic reviews	49	49	0
Probiotics in infants for prevention of allergic disease and food hypersensitivity.	Cochrane database of systematic reviews	5	5	0
Neuraminidase inhibitors for preventing and treating influenza in healthy adults and children	Cochrane database of systematic reviews	312	385	73
Tamiflu report comes under fire.	Nature	101	129	28

Kangaroo mother care to reduce morbidity and mortality in low birthweight infants.	Cochrane database of systematic reviews	11	21	10
Skin-to-skin care for procedural pain in neonates.	Cochrane database of systematic reviews	69	81	12
Different infusion durations for preventing platinum-induced hearing loss in children with cancer	Cochrane database of systematic reviews	10	16	6
Interventions for promoting participation in shared decision-making for children with cancer	Cochrane database of systematic reviews	12	18	6
Minimally invasive surgery versus open surgery for the treatment of solid abdominal and thoracic neoplasms in children	Cochrane database of systematic reviews	8	14	6
Treatment including anthracyclines versus treatment not including anthracyclines for childhood cancer	Cochrane database of systematic reviews	8	14	6
Oral antihistamine-decongestant-analgesic combinations for the common cold.	Cochrane database of systematic reviews	4	26	22
Medical interventions for the prevention of platinum-induced hearing loss in children with cancer	Cochrane database of systematic reviews	10	15	5
Vitamin C for preventing and treating the common cold	Cochrane database of systematic reviews	295	348	53
Zinc for the common cold	Cochrane database of systematic reviews	271	311	40
Sucrose for analgesia in newborn infants undergoing painful procedures (2013)	Cochrane database of systematic reviews	42	46	4
Clinical Practice Guideline: The Diagnosis, Management, and Prevention of Bronchiolitis	Pediatrics	174	208	34
Treating cough and cold: Guidance for caregivers of children and youth.	Paediatrics and Child Health	0	27	27
Antibiotic Treatment of Children With Sore Throat	JAMA: Journal of the American Medical Association	0	9	9
Dietary exclusions for established atopic eczema.	Cochrane database of systematic reviews	3	8	5
Will Tamiflu recommendations change this winter?	British Medical Journal (Clinical Research Edition)	101	106	5
Vaccines for measles, mumps and rubella in children	Cochrane database of systematic reviews	198	230	32
Probiotics for treating acute infectious diarrhoea	Cochrane database of systematic reviews	17	24	7

Breastfeeding or breast milk for procedural pain in neonates.	Cochrane database of systematic reviews	10	14	4
Early skin-to-skin contact for mothers and their healthy newborn infants	Cochrane database of systematic reviews	66	86	20
Interventions for improving communication with children and adolescents about their cancer	Cochrane database of systematic reviews	11	17	6
Neuraminidase inhibitors for preventing and treating influenza in healthy adults	Cochrane database of systematic reviews	16	19	3
The Tamiflu trials.	British Medical Journal (Clinical Research Edition)	191	198	7
Sweet-tasting solutions for needle-related procedural pain in infants one month to one year of age.	Cochrane database of systematic reviews	47	51	4
Oral zinc for treating diarrhoea in children	Cochrane database of systematic reviews	19	24	5
Sweet tasting solutions for reduction of needle-related procedural pain in children aged one to 16 years	Cochrane database of systematic reviews	14	17	3
The common cold.	The Lancet (British Edition)	1	4	3
Honey for acute cough in children	Cochrane database of systematic reviews	36	71	35
Inhaled corticosteroids in children with persistent asthma: effects on growth.	Cochrane database of systematic reviews	158	173	15
Physical training for cystic fibrosis	Cochrane database of systematic reviews	1	4	3
Graduated driver licensing for reducing motor vehicle crashes among young drivers	Cochrane database of systematic reviews	2	10	8
Inhaled corticosteroids in children with persistent asthma: dose response effects on growth	Cochrane database of systematic reviews	102	112	10
Interventions for preventing unintended pregnancies among adolescents	Cochrane database of systematic reviews	4	11	7
Interventions for preventing obesity in children	Cochrane database of systematic reviews	104	140	36
Lactose avoidance for young children with acute diarrhoea	Cochrane database of systematic reviews	57	65	8
Oral antihistamine-decongestant-analgesic combinations for the common cold: do they work?	Evidence-Based Child Health: A Cochrane Review Journal	6	9	3

The Cochrane Library and safety of regular long-acting beta2-agonists in children with asthma: an overview of reviews	Evidence-Based Child Health: A Cochrane Review Journal	0	17	17
Scared Straight' and other juvenile awareness programs for preventing juvenile delinquency (2002)	Cochrane database of systematic reviews	40	43	3
Self-management education for cystic fibrosis	Cochrane database of systematic reviews	3	4	1
Does This Patient Have Strep Throat?	JAMA: Journal of the American Medical Association	0	9	9
The Cochrane Library and procedural pain in children: an overview of reviews	Evidence-Based Child Health: A Cochrane Review Journal	1	22	21
The prevention of eczema in infants and children: an overview of Cochrane and non-Cochrane reviews	Evidence-Based Child Health: A Cochrane Review Journal	9	12	3
Interventions for promoting physical activity in people with cystic fibrosis	Cochrane database of systematic reviews	17	18	1
The Cochrane Library and the Treatment of Sore Throat in Children and Adolescents: An Overview of Reviews	Evidence-Based Child Health: A Cochrane Review Journal	5	10	5
Scared Straight" and other juvenile awareness programs for preventing juvenile delinquency (2013)	Cochrane database of systematic reviews	32	31	-1
			Average:	11.0491803
			Median:	5
